# The role of oxidative stress in patients with recurrent pregnancy loss: a review

**DOI:** 10.1186/s12978-021-01257-x

**Published:** 2021-10-16

**Authors:** Vjosa A. Zejnullahu, Valon A. Zejnullahu, Ernad Kosumi

**Affiliations:** 1 Department of Obstetrics and Gynecology, University Clinical Center of Kosovo, 10000 Prishtina, Kosovo; 2 Faculty of Medicine, University of Prishtina “Hasan Prishtina”, Prishtina, Kosovo; 3 Department of Abdominal Surgery, University Clinical Center of Kosovo, 10000 Prishtina, Kosovo

**Keywords:** Recurrent pregnancy loss, Oxidative stress, Oxidative stress biomarkers

## Abstract

**Background:**

Recurrent pregnancy loss (RPL) presents one of the main problems in the field of reproductive medicine, due to the unknown etiology in 50% of cases, as well as limited evidence-based diagnostic and therapeutic modalities. Recent studies indicate that systemic and placental oxidative stress (OS) represents an essential factor in the etiopathogenesis of RPL. This article is a comprehensive narrative synthesis of previously published studies concerning the role of oxidative stress in the etiology of recurrent pregnancy loss.

**Methods:**

We conducted literature search of published studies in the English language focusing on oxidative stress and its association with recurrent pregnancy loss (RPL) utilizing the Medline and Cochrane databases from 2000 through January 2021. The keywords used were “recurrent pregnancy loss” “oxidative stress and recurrent pregnancy loss” and “oxidative stress biomarkers and recurrent pregnancy loss”.

**Results:**

The search yielded 1116 publications, of which 92 were included in the final analysis. Reactive oxygen species (ROS) and reactive nitrogen species (RNS) at basal levels have various physiological functions whereas deviation from redox window is associated with different pathologies including early pregnancy loss. The currently available studies support the concept that increased oxidative stress (OS) and deficient antioxidant protection is implicated in the etiology of recurrent pregnancy loss (RPL) but underlying mechanisms through which OS affects pregnancy outcome remains largely indefinable.

**Conclusions:**

Future research in this field can provide new insights regarding the OS-mediated damage in recurrent pregnancy loss as well as potential applications of antioxidant therapy in this group of patients.

## Plain Language summary

Recurrent pregnancy loss is a significant health problem in reproductive medicine due to the idiopathic etiology in 50% of cases and limited evidence-based practice. The role of oxidative stress in recurrent pregnancy loss (RPL) is an understudied and enthralling field of investigation. We reviewed the existing information based on the published literature on this complex topic and found that oxidative/nitrosative stress complemented with inefficient antioxidant capacity presents an essential factor in the etiology of recurrent pregnancy loss (RPL). However, based on the currently available knowledge underlying mechanisms through which OS-imbalance affects the increased risk of RPL remains unclear. Future research with a larger number of patients, can provide new insights regarding the molecular mechanisms as well as modifiable factors to decrease OS in this clinical entity.

## Background

By definition a pregnancy loss includes all pregnancy losses from the time of conception until 24 weeks of gestation or before the fetus reaches viability. Recurrent pregnancy loss (RPL), defined as three or more consecutive pregnancy losses, affects 0.5–2% of women in childbearing age [1].

According to the consensus statement released by the European Society of Human Reproduction and Embryology (ESHRE), recurrent pregnancy loss describes 2 ore more pregnancy losses, diagnosed by serum or urine human chorionic gonadotropin. Diagnosis of RPL includes the spontaneous demise of a pregnancy after spontaneous conception and after assisted reproductive technology (ART) and excludes ectopic and molar pregnancies [1].

RPL is one of the main problems in the field of reproductive medicine and presents an emotionally stressful experience for affected couples.

Advanced maternal age, gestational age of prior pregnancy loss and its cause are among key factors in recurrent pregnancy loss. Other known risk factors of RPL include acquired and congenital uterine abnormalities, leiomyoma, intrauterine adhesions, defective endometrial receptivity, antiphospholipid syndrome (APS), diabetes mellitus, polycystic ovary syndrome, hypo or hyper-thyroidism, thyroid autoimmunity, hyperprolactinemia, genetic factors and sperm DNA fragmentation [2–5]. There is inconclusive evidence regarding the association between recurrent pregnancy loss (RPL) and obesity, smoking, alcohol, caffeine consumption and exposure to environmental chemicals [6, 7].

Recent studies highlight the role of oxidative stress and oxidative biomarkers in the pathophysiology of recurrent pregnancy loss [8, 9].

The main objective of this review was to investigate the association between oxidative/nitrosative stress and recurrent pregnancy loss and to summarize existing information based on the published literature. Furthermore, the authors aim to present objective conclusions based upon the literature reviewed and identify gaps and limitations to provide a motivation for future research.

## Methods

This article is a narrative overview of some original studies and review papers regarding the role of oxidative stress in pregnancy outcome, particularly focusing on oxidative stress and its association with recurrent pregnancy loss (RPL). By-products of oxygen metabolism, physiological functions of the free radicals and antioxidant mechanisms are concisely explained.

### Identify research question

The main research question in this article is: Does oxidative/nitrosative stress have a major role in the etiology of RPL and does the activity of oxidants/antioxidants changes in normal pregnancy and recurrent pregnancy loss?

### Search strategy

Medline database was searched using the terms “recurrent pregnancy loss”, “oxidative stress and recurrent pregnancy loss” and “oxidative stress biomarkers and recurrent pregnancy loss” from 2000 through January 2021. In addition, literature search was conducted utilizing the Cochrane database. We conducted an electronic and a manual search of cross-references and included published studies in the English language concerning the involvement of oxidative stress (OS) in RPL.

### Study selection

Literature related to the focused purpose of the study and studies published during the outlined time frame were included.

Studies assessing recurrent pregnancy loss, oxidative stress and prooxidants/antioxidant systems were eligible for inclusion. The first screening of publications was carried out based on the titles and abstracts. The remaining publications were assessed using the full-texts. Criteria used to exclude ineligible papers were: studies published in language other than English, comments, personal opinions, old data, not relevant to the topic, no quantifiable evidence, non-relevant population and outcome.

### Data extraction

Data extraction was performed from the abstracts and full text and consisted of following information authors, publication year, research questions, study design and objectives, study outcome and recommendations.

## Results

### Search flow

**Fig. 1:**
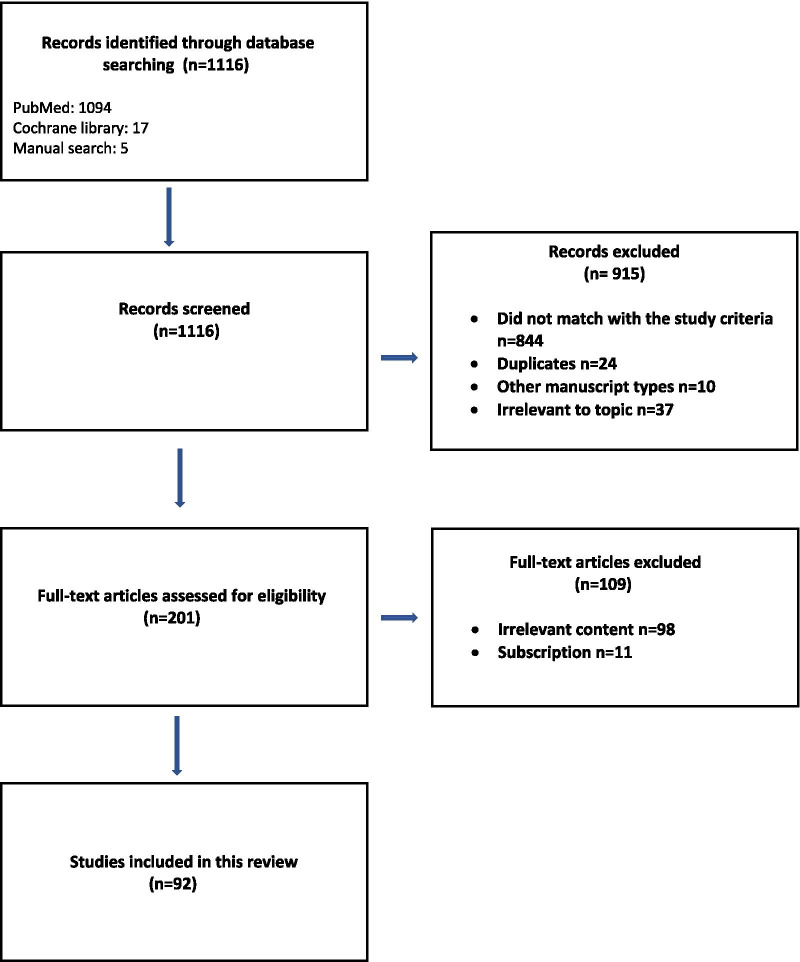
Flow diagram of narrative review of literature

From the original search reviewers identified 1116 records through database searching of which 915 were excluded with reason, did not match with the study criteria, duplicates, other manuscript types, irrelevant to topic. The remaining 201 articles met the criteria for full-text review with another 109 being excluded with reason, irrelevant content and subscription. Finally, 92 articles fulfilled the inclusion criteria and were included in the current unsystematic narrative review. Details of the search strategy are provided in Fig. 1.

## Main text

### By-products of O_2_ metabolism, detoxification mechanisms and the role of oxidative stress

Mitochondrial oxidative phosphorylation is the final stage of cellular respiration, following glycolysis, pyruvate oxidation and the citric acid cycle. During oxidative metabolism, O_2_ is employed as the terminal electron acceptor for mitochondrial electron transport chain (ETC). In the mitochondrial matrix O_2_ is reduced into H_2_O by the respiratory chain. However, one-electron reduction of molecular oxygen (O_2_) which happens because of electron leakage through respiratory chain complex I and III, will generate highly reactive molecules known as reactive oxygen species (ROS). Therefore, free radicals derived from oxygen (ROS) are a product of aerobic cellular respiration [10, 11].

The most common toxic radical species include superoxide anion, oxygen radical, hydroxyl radical, alkoxy-radical, peroxyl radical, nitric oxide and nitrogen dioxide [12, 13]. The non-radical species such as hydrogen peroxide, hypochlorous acid, hypobromous acid and other species, can induce cellular damage after being converted into more aggressive radical species [13, 14]. The most important ROS derived from enzymatic and by a non-enzymatic reaction is superoxide anion [15]. Superoxide anion is rapidly dismutated to H_2_O_2_ either in the matrix by manganese superoxide dismutase (MnSOD) or in the intermembrane space by Cu/ZnSOD. Hydrogen peroxide (H_2_O_2_) is further degraded to O_2_ and H_2_O by antioxidant enzymes as catalase (CAT) and glutathione peroxidase (GPx). Reduced glutathione (GSH) acts as peroxide scavenger and its oxidised form (GSSG) can be further reduced by glutathione reductase (GR) using NADPH as substrate [16]. H_2_O_2_ is a key messenger molecule in redox signaling but at higher concentrations can induce oxidative damage [16, 17]. The mitochondria, peroxisomes, endoplasmic reticulum, endothelial cells, neutrophils, macrophages, xanthine oxidoreductase and myeloperoxidase are endogenous source of ROS generation [18–20]. In addition, the exogenous sources of ROS production include air pollution, alcohol, tobacco smoke, heavy metals, industrial solvents, pesticides, gamma and UV radiation as well as certain drugs [21]. Aerobic eukaryotes have developed antioxidant defense mechanisms which act by mitigating damaging effects of excessive ROS production, hence under physiological conditions there is a dynamic balance between the ROS generation and elimination [22]. The oxidative stress is a consequence of excessive ROS production, reduced antioxidant capacity and mitochondrial dysfunction [22, 23]. When oxidative stress (OS) occurs, macromolecular homeostasis can be substantially affected because of lipid peroxidation, protein modifications and DNA oxidation by free radicals [23–25].

At physiological levels ROS modulate cell cycle and proliferation, activate angiogenesis, enable phagocytosis, activate antioxidant genes and pro-inflammatory cytokines [22, 24, 26, 27]. Low, physiological levels of ROS are necessary for self-renew of stem cells while increased ROS levels activate proliferation, differentiation, senescence and apoptosis of stem cells in concentration-dependent manner [28]. Detrimental effects on living organisms arise when ROS, exceed basal levels needed for cell signaling and transduction [22]. Therefore, the beneficial effects of ROS are achievable within physiological levels and deviation from redox window (as oxidative or reductive stresses) [29] will be associated with different pathologies including neurodegenerative diseases [30] diabetes [31] cardiovascular disease and atherosclerosis [32] rheumatoid arthritis [33] respiratory disease [34] carcinogenesis [35] ageing [36] female reproductive disease, pregnancy complications and recurrent pregnancy loss [37].

### Oxidative stress (OS) and oxidative biomarkers in recurrent pregnancy loss (RPL)

Oxidative biomarkers are important tool in measuring oxidative stress in clinical samples in different pathologies. Common biomarkers measured in oxidative stress research include hydrogen peroxide (H_2_O_2_), hydroxyl radicals (OH^−^), peroxyl radicals (ROO^−^), protein carbonyl content (PC) as a marker of oxidative modifications of proteins, malondialdehyde (MDA) as a marker of ROS-mediated damage of membrane lipids, 8-hydroxyguanosine (8-OHG) as a RNA damage product and 8-hydroxydeoxyguanosine (8-OHdG) as a biomarker of oxidative DNA damage [38].

Early pregnancy is characterized by increased level of polymorphonuclear leukocyte count, which in turn contribute to the oxidative stress because of superoxide generation from these primed leucocytes [39].

In women with recurrent abortions increased generation of radical species from leukocytes was demonstrated via increased granulocyte spontaneous chemiluminescence when compared to the reference group [40].

NADPH-oxidase found in polymorphonuclear leukocytes, present a major source of superoxide generation in early and term pregnancy [41, 42]. NADPH oxidases (Nox) are a family of isoenzymes found in neutrophils, vascular smooth muscle cells and placenta [43, 44]. Placental isoform or human placental NADPH oxidase has different properties compared to neutrophils and macrophages [43].

Evidence suggest that NADPH oxidase is the main source of superoxide generation before 10 weeks of gestation when chorionic villi are exposed to relative hypoxia [41, 45].

A study investigating NADPH oxidase activity and antioxidant capacity in placental tissues from early and term pregnancies showed corresponding increase of antioxidant capacity and Nox activity emphasizing the role of superoxide production in modulation of antioxidant defense in early pregnancy [42].

Altered balance between oxidants and antioxidants can trigger oxidative stress in human placenta and has important implications in the etiology of RPL.

Studies investigating oxidants, consistently reported increased levels in plasma and placental tissues of recurrent miscarriage patients. Superoxide anion radical (SOA) is the most widespread ROS and an imbalance in the homeostatic concentrations of superoxide anion and H_2_O_2_ can lead to the production of hydroxyl ions. Significance of this highly reactive free radical is that can cause severe cell injury by reacting with organic and non-organic molecules [16]. A recent study reported significant increase of SOA in plasma samples of RPL patients compared to the control samples (45.2 ± 6.10 nmol/ml vs 35.3 ± 5.45 nmol/ml). Similar difference was observed for the placental tissue SOA between the two groups (5.93 ± 0.78 µmol/min/mg in RPL patients and 4.67 ± 0.62 µmol/min/mg in healthy pregnant women) [8].

Another parameter of oxidative stress assayed in plasma and placental tissue is H_2_O_2_. Two studies assessing this biomarker yielded similar results. Ghneim et al., reported increased levels of plasma H_2_O_2_ in RPL group compared to healthy women and non-pregnant women (7.4 ± 0.70 pM; 6.70 ± 0.70 pM; 6.20 ± 0.70 pM; respectively) [9]. Comparably, Al-Sheikh and coauthors found increased levels of this marker in placental tissues of recurrent miscarriage patients 3.38 ± 0.46 nmol/min/mg in RM group and 2.41 ± 0.35 nmol/min/mg in healthy pregnant subjects [8].

Importantly, increased levels of SOA and H_2_O_2_ in both studies were associated with depletion of enzymatic antioxidants such as superoxide dismutase (SOD) catalase (CAT), glutathione reductase (GR) and glutathione peroxidase (GPx) as well as decreased expression of all examined antioxidant genes (GPx, GR, SOD and CAT) [8, 9].

The superoxide dismutase (SOD) is one of the main antioxidant enzymes and its activity is regarded as a first line of defense in preventing oxidative stress (OS). There are three isoforms of SOD including Cu/ZnSOD defined as SOD1, MnSOD or SOD2 and extracellular enzyme, or SOD3. A study conducted to examine SOD1 and SOD2 activities in samples of RPL patients and controls found interesting results. SOD1 plasma activity was 5.43 ± 0.69 nmol/min/ml in recurrent miscarriage group and 6.11 ± 0.73 nmol/min/ml in healthy controls. Similar results were observed for SOD2 plasma activity in study and control group (4.50 ± 0.55 nmol/min/mg vs 5.12 ± 0.66 55 nmol/min/mg) [46]. Furthermore, intracellular hsSOD1 transcripts were downregulated by 54% in placental tissue of women with RPL. In this study increased levels of SOA were associated with concurrent decrease of SOD1 and SOD2 activity in plasma and placental tissues of women with RPL [46].

Yiyenoglu et al., conducted a prospective controlled study to evaluate oxidative stress markers and observed increased oxidative stress index levels and decreased antioxidant capacity in women with a history of RPL [47].

Therefore, oxidative stress associated with deficient antioxidant defense is regarded as one of the key factors in the etiopathogenesis of RPL.

It should be emphasized that reactive oxygen species (ROS) and reactive nitrogen species (RNS) can act together to induce cellular injury. Nitric oxide (NO) is nitrogen based-reactive species and as membrane permeable radical can react with superoxide to form peroxynitrite, an agent with oxidizing and nitrating properties [48]. Therefore, NO can mediate ROS-induced lipid peroxidation (LPO). In particular polyunsaturated fatty acids (PUFA) are less resistant to free-radical attack because of the abundance of double bonds in their structure [49]. Malondialdehyde (MDA) is toxic end-product of autoxidation of polyunsaturated fatty acids (PUFA) and important index of oxidative damages. As a result of lipid peroxidation several membrane functions are disturbed including membrane permeability, fluidity and enzyme activity [48].

Studies evaluating the level of oxidative stress by assessing malondialdehyde (MDA) as a product of lipid peroxidation, have found elevated plasma and placental levels of this marker in patients with recurrent pregnancy loss (RPL). Reported plasma MDA levels were 5.95 ± 0.91 pM for recurrent miscarriage patients and 5.12 ± 0.81 pM for healthy controls [9]. Similarly in another study, placental MDA levels were highly significantly increased in recurrent miscarriage subjects compared to the controls (334 ± 45.8 nmol/g wet weight versus 258 ± 35.7 nmol/g wet weight) [8].

A case–control study investigating the MDA levels in RPL patients, healthy pregnant and non-pregnant women found a significant elevation of this marker in the study group. Additionally a highly significant difference was observed in the MDA/vitamin E ratio between recurrent miscarriage group and healthy controls [50].

El-Far et al., investigated MDA levels in women with idiopathic recurrent abortions. They reported significantly increased MDA levels and NO production in the study group compared to the control groups (p < 0.05 for each comparison). The authors conclude that possible oxidative damage due to the increased generation of oxidative species and diminished antioxidative defense may be responsible for recurrent abortions [51].

Although, mechanism by which oxidative stress (OS) contribute to anticardiolipin antibodies (aCL) formation is not fully clarified, a study conducted by Ferro et al., demonstrated a mean decrease in aCL titer and reduced rate of thrombin generation in patients treated with non-enzymatic antioxidants. In this study all patients treated with antioxidants showed a significantly increased plasma levels of vitamin C and vitamin E as well as a mean decrease in aCL titer by 61% (range 16–84%) [52].

Another study evaluating extent of somatic DNA damages and oxidative stress conditions found a statistically significant increase in the mean micronuclei frequency (MN). MDA levels were significantly increased in women with recurrent pregnancy loss when compared to the control group (1.63 and 0.66 respectively) [53]. Collectively, these studies highlight the role of oxidative stress (OS) in recurrent pregnancy loss (RPL).

### Antioxidant defense mechanisms and recurrent pregnancy loss (RPL)

Because generation of reactive oxygen species (ROS) is the result of the aerobic cellular respiration and ATP production, living systems have developed necessary defense mechanisms against oxygen toxicity to maintain a delicate homeostatic balance between oxidant and antioxidant state. Antioxidant defense system involves enzymatic and non-enzymatic agents. Endogenous enzymatic antioxidants are the first line of defense and include matrix manganese superoxide dismutase (MnSOD), intermembrane cooper/zinc superoxide dismutase (Cu/ZnSOD), glutathione reductase (GR), glutathione peroxidase (GPx), and catalase (CAT). Ceruloplasmin, transferrin, ferritin and albumin are non-enzymatic antioxidants in the blood plasma [54]. Natural non-enzymatic antioxidants are represented by vitamin A, vitamin E, vitamin C, polyphenols, uric acid, flavonoids, carotenoids, glutathione, bilirubin and melatonin [55]. Metallothionein has antioxidant properties because of the presence of the thiol groups (–SH) and melatonin is effective hydroxyl radical scavenger [54]. Authors investigating antioxidant enzyme activity in the placenta, demonstrated strong correlation of antioxidant activity with gestational age and oxygen concentration within the placental tissues [56]. Data from this study revealed increased activity and increased expression in the mRNA concentration of the antioxidant enzymes CAT, GSH and Cu/ZnSOD with gestational age and with the oxygen tension in the intervillous space [56]. Thus the ensuing conclusion was that at the end of first trimester, establishment of maternal circulation is associated with burst of oxidative stress even in normal pregnancy and has important role in normal placentation [56]. Increased oxygen concentration and diminished antioxidant capacity will result in impaired or abnormal placentation and early pregnancy failure. In normal pregnancies intervillous circulation is initiated in the peripheral regions of the placenta and is fully established during the second trimester. In abnormal pregnancies, premature onset of the maternal placental circulation secondary to reduced trophoblastic invasion, induces oxidative damage to the villous trophoblast and is a key factor in early pregnancy loss [57]. Association of miscarriage and pregnancy with increased oxidative stress was further supported with the results reported by Jenkins et al. In this study normal term pregnancies were associated with increased superoxide dismutase (SOD) levels early in the first trimester while miscarriage group had significantly reduced levels of SOD [58]. Glutathione (GSH) is hydrosoluble antioxidant and comprises three amino acids: glycine, cysteine and glutamic acid. It functions in conjunction with glutathione peroxidase (GPx), glutathione reductase (GR) and glutathione oxidase (GOx) [55]. Glutathione (GSH) and glutathione peroxidase (GPx) are efficient protective antioxidants responsible to maintain redox homeostasis [59]. The activity of GPx depends on the presence of reduced glutathione (GSH), which is major cellular redox buffer in cells and it is oxidized by GPx. Oxidized glutathione (GSSG) is reduced back to GSH by glutathione reductase (GR). Thus, the reducing intracellular environment is maintained by the GSH/GSSG redox couple to ensure low physiological ROS levels [59]. A study evaluating enzymatic antioxidant levels in healthy pregnant women, non-pregnant controls and women with recurrent pregnancy loss (RPL) demonstrated significantly decreased glutathione (GSH) and glutathione reductase (GR) levels, low GSH/GSSG ratio and increased oxidized glutathione (GSSG) levels in plasma and placental tissues of women with RPL compared to healthy controls. Reported plasma glutathione reductase (GR) activity was 1.59 ± 0.20 U/l for RPL patients and 1.83 ± 0.26 U/l for healthy pregnant controls. Authors reported a similar reduction of placental GR activity in the RPL group when compared to the healthy controls (0.36 ± 0.05 U/mg protein against 0.75 ± 0.10 U/mg protein). Placental tissue glutathione (GSH) levels were also significantly decreased in the recurrent miscarriage group compared to the control group (5.80 ± 0.77 nmol/mg tissue vs 6.70 ± 0.71 nmol/mg tissue). Additionally, in this study plasma GSH/GSSG ratio was 52.5 ± 6.77 in non-pregnant controls, 48.3 ± 5.99 in healthy pregnant women and 42.1 ± 5.86 in recurrent pregnancy loss patients [9]. Considering the role of GSH as a ROS scavenger GSH/GSSG ratio is important biomarker of oxidative damage [60]. Al sheik et al., examined levels of enzymatic antioxidants in non-pregnant, healthy pregnant and RPL women. Plasma glutathione peroxidase (GPx) levels in RPL patients and healthy pregnant women (HP) were 0.86 ± 0.10 nmol/min/ml and 1.06 ± 0.13 nmol/min/ml, respectively). Similar pattern was observed for placental tissue GPx in RPL group and healthy controls (0.28 ± 0.04 µmol/min/mg protein and 0.36 ± 0.04 µmol/min/mg protein, respectively). Placental tissue of RPL patients revealed highly significant decreased levels of catalase (CAT) (0.70 ± 0.10 µmol/min/mg protein in RPL group and 0.91 ± 0.12 µmol/min/mg protein in healthy pregnant group). Decreased activity of enzymatic antioxidants GPx and CAT was associated with low GSH/GSSG ratio in the study group [8]. The glutathione S-transferase (GST) families are believed to exert a critical role in protection against OS by detoxifying DNA, catechol products and oxidized lipids, generated as a result of oxidative damage [61]. GST izoenzymes catalyze conjugation of reduced glutathione and act as Selenium-independent GSH peroxidases against organic hydroperoxides [62]. Studies investigating the relation between the RPL and gene polymorphisms of glutathione S-transferase (GST) M_1_ and T_1_ suggest increased risk of RPL in women with GSTM1 null polymorphism [63]. Therefore, administration of *N*-acetylcysteine (NAC), a source of sulfhydryl (SH) groups and acetylated precursor of reduced glutathione, appears beneficial in oxidative stress conditions associated with decreased GSH. Evidence suggest clinical benefits of NAC use, in management of idiopathic RPL [64]. Micronutrients selenium (Se), zinc (Zn), cooper (Cu) and manganese (Mn) are cofactors for the enzymatic antioxidants and have a crucial role in antioxidant defense and ROS scavenge. Zinc, magnesium, cooper and selenium deficiency are associated with pregnancy complications, preeclampsia, premature delivery, fetal growth retardation and low birth weight [65]. Selenium is incorporated into the glutathione peroxidase, thioredoxin reductases and selenoprotein-P [66]. Decreased levels of selenium and GPx were previously reported by Desai et al. In this study red cell selenium levels were 88.69 ± 11.15 ng/ml for the miscarriage group and 96.96 ± 8.87 ng/ml for the healthy pregnant group [67]. A pilot study conducted in India yielded comparable results as the mean red cell selenium levels were significantly lower in the RPL patients (119.55 ± 32.94 ng/ml) compared to the control group (150.85 ± 37.63 ng/ml) [68]. Selenium deficiency is associated with reduced antioxidant capacity of GPx [69]. Ghneim et al., found decreased selenium levels in plasma samples of RPL women (75.3 ± 11.2 µg/l for the recurrent miscarriage group and 87.6 ± 12.6 µg/l for the healthy pregnant women). Similarly the placental tissue selenium levels were significantly lower compared to the control group (9.67 ± 1.32 nmol/g versus 15.5 ± 1.91 nmol/g) [9]. Yet, literature is inconclusive when it comes to the role of decreased Selenium levels in the etiology of RPL since several studies evaluating Se concentrations reported conflicting results. Al-Kunani and coauthors observed significant reduction of the mean hair selenium levels in the recurrent miscarriage group compared with the control group (0.14 µg/g vs 0.34 µg/g) but failed to show this difference in serum samples [70]. Similar results were reported by Thomas et al., were median selenium content was 0.80 ppm for non-pregnant women with a history of 2 or 3 recurrent miscarriages and 0.68 ppm for the non-pregnant women with uncomplicated obstetric history. The authors concluded that selenium supplementation in RPL cannot be recommended since the importance of selenium deficiency in recurrent miscarriage has still not been determined [71]. Zinc is an essential cofactor for Cu/Zn superoxide dismutase and acts as an antioxidant mitigating oxidative stress, has important role in the regulation of GPx and expression of metallothionein and importantly, inhibits NADPH oxidases. In deficient or excessive levels it can also act as an prooxidant [72]. Role of decreased cellular Zn concentration in oxidative stress is not fully clarified and is mainly attributed to the decreased activity of Cu/Zn SOD and ceruloplasmin. However evidence suggest that there is no positive correlation between Cu/Zn activity and tissue zinc concentration or dietary intake [73]. Current data indicate significantly decreased plasma zinc levels in RPL subjects compared to healthy controls (2.84 ± 0.36 µmol/l vs 3.55 ± 0.49 µmol/l) [8]. Albeit, the existing results highlight the significance of deficient antioxidant defense in the etiology of RPL, the role of micronutrients as cofactors of enzymatic antioxidants in RPL patients’ needs further investigation in future randomized controlled clinical trials. Alpha-tocopherol, ascorbic acid, beta carotene, taurine, transferrin, ferritin and ceruloplasmin have antioxidant properties [55]. Ceruloplasmin and transferrin act by sequestering free iron ions and inhibit hydroxyl radical (OH) production in the Fenton reaction [74]. Ferritin is important to maintain intracellular iron balance and acts by binding free iron [54]. Results from a recent study indicate that magnesium (Mg) value cut-off levels at ≤ 0.81 demonstrated high sensitivity and specificity (80% and 68.6%, respectively) in the detection of women with recurrent miscarriage [75].

Whereas vitamin C (ascorbic acid) and vitamin E (alpha-tocopherol) have important antioxidant properties, Cochrane database systematic review on effectiveness and safety of any vitamin supplementation on the risk of miscarriage, found that antioxidant vitamin supplementation had no effect on early or late miscarriage [76].

### Angiogenesis and apoptosis-related genes and recurrent pregnancy loss (RPL)

Another emerging field of investigations in the etiology of RPL include angiogenesis and apoptosis-related genes. Studies investigating these genes suggest important role of OS-mediated angiogenesis in aberrant placentation and recurrent pregnancy loss [77, 78]. Human placenta has a hemochorial type of placentation. Vascular and embryonic morphogenesis are a prerequisite for normal development of uteroplacental and fetal circulation [79]. Vascular endothelial growth factor (VEGF) and fibroblast growth factor (FGF) with their respective receptors are major angiogenic factors in fetal and placental angiogenesis [80]. During early pregnancy expression of VEGF mRNA is greater in fetal placental tissue compared with maternal endometrial tissue. Consequently, inappropriate expression of angiogenic factors may contribute to the impaired placental vascularization and placental dysfunction and appears to be involved in the etiology of infertility and fetal growth retardation [80]. Angiogenesis is regulated by proangiogenic and antiangiogenic factors and recent evidence indicate that oxidative stress is involved in physiological and pathological angiogenesis. VEGF-dependent and VEGF-independent signaling pathways of angiogenesis are mediated by oxidative stress [81, 82]. Association of RPL with aberrant expression of angiogenesis and apoptosis-related genes was previously reported in several studies [77, 78]. Choi et al., reported decreased expression of 6 angiogenesis-related genes and increased expression of 12 apoptosis-related genes in chorionic villi from the patients with recurrent pregnancy loss when compared to the normal control group [78]. Zhu et al. found that microRNA-16 (miR-16) expression is upregulated in villi and decidua of recurrent pregnancy loss patients. miR-16 overexpression inhibits placental angiogenesis via VEGF suppression and plays important role in the pathogenesis of RPL [83]. Aberrant angiogenesis in RPL may be related to VEGF dysregulation including VEGF gene polymorphisms [84]. He et al., found significantly decreased mRNA and protein level in the villi and decidua from women with RPL compared to the normal early-pregnancy group [77]. Albeit, the direct relationship between the VEGF and RPL was not established, evidence suggest that reduced VEGF expression in RPL patients can contribute to poor angiogenesis and is related to RPL occurrence [77]. In a case control study, authors reported lower relative expression of VEGF gene and increased expression of VEGF receptors (VEGFR1 and VEGFR2) in the endometrium of women with idiopathic recurrent pregnancy loss compared to the control group. Also, study group with RPL showed significantly increased serum levels of VEGF when compared to the healthy controls (27.87 ± 7.42 pg/ml vs 10.20 ± 2.81 pg/ml). The increased expression of VEGF receptors may act as a compensatory mechanism for the decreased VEGF expression, a speculation which warrants further investigation [85].

In women with RPL levels of soluble VEGF receptor-1 (sFlt-1) and VEGF mRNA in the chorionic samples are significantly increased compared to the control group hence, suggesting that overexpression of VEGFA and FLT1 genes is implicated in the etiology of early recurrent pregnancy loss [86].

### Nitrosative stress (NS) and recurrent pregnancy loss

The increased body of evidence associates nitrosative stress with recurrent pregnancy loss (RPL). Reactive nitrogen species (RNS) are nitrogen-containing compounds including nitric oxide (NO), nitrous oxide (N_2_O), peroxynitrite (NO3^−^), nitroxyl anion (NO^−^), and peroxynitrous acid (HNO_3_) [52]. Nitric oxide (NO) is membrane-soluble free radical synthetized by nitric oxide synthase (NOS) and demonstrates pathological effect at higher concentrations, in particular when reacting with superoxide to form peroxynitrite. Peroxynitrites are potentially hazardous because they cause nitrosative modifications of proteins and lipids. Excessive production of RNS beyond biological system’s ability to eliminate them causes nitrosative stress that act synergistically with oxidative stress and causes ROS/RNS-induced injury to the cells [48]. Evidence suggest the role of nitrosative stress in RPL patients which is revealed by increased NO levels. A retrospective case–control study Raffaelli et al., showed a significant increase in platelet NO in recurrent pregnancy loos patients (RPL) compared to healthy pregnant women [87]. Genetic polymorphisms in the NOS2 promoter, altered gene expression and excessive generation of nitric oxide (NO) was reported as a risk factor for RPL. In a case–control study evaluating 149 women with recurrent miscarriage, authors showed that presence of variant T(TT/GT) of rs2779249(-1290G>T) of NOS2 was significantly associated with RPL compared to controls, even after adjustments for ethnicity, number of pregnancies and alcohol consumption [88]. During normal pregnancy NO is involved in maternal vasodilatation, trophoblast invasion, apoptosis and platelet aggregation in the intervillous space [89]. Yet, NO overproduction will cause p53 protein phosphorylation, apoptosis and impaired placental proliferation [90]. Endothelium-derived NO act as a physiological mediator of early pregnancy and is involved in the regulation of placental function. Several studies support the concept of polygenetic etiology of RPL. A prospective case–control study investigated 105 women with idiopathic recurrent miscarriage compared to 91 healthy controls and reported a 1.6-fold increased risk of recurrent miscarriage in heterozygous carriers of the NOS3 polymorphism. Although, the association between recurrent miscarriage and NOS3 polymorphism was demonstrated, authors could not specify at what level a lack of eNOS derived NO affects the risk of RPL [91].

A systematic review and meta-analysis conducted by Su et al., found significant association of VEGF (21154G>A), p53 (codon 72) and eNOS (Glu298Asp) gene polymorphisms with recurrent pregnancy loss thus, suggesting functional consequences of these polymorphisms in the etiology of RPL, notably with various effects in different populations.

Consequently, VEGF (-1154G>A), p53 (codon 72) and eNOS (Glu298Asp) polymorphisms indicate a potential markers to identify women at increased risk of RPL in clinical management [92].

In general, these findings illustrate complexity associated with the contribution of oxidative stress and apoptosis/angiogenesis-related genes in early pregnancy loss and recurrent pregnancy loss.

## Conclusions

This integrative overview based on the results of previous studies has shown that oxidative and nitrosative stress play an important role in the etiopathogenesis of early pregnancy loss and recurrent pregnancy loss. Free radicals, reactive oxygen species (ROS) and reactive nitrogen species (RNS) are a product of aerobic metabolism. At basal or moderate levels these species have various physiological functions. Cellular oxidants are involved in the maintenance of redox homeostasis which is necessary for normal physiological functions. They promote natural defenses, contribute to the proliferation and apoptosis depending on signaling and executive pathways, regulate immune response, activate proliferative pathways and exert signaling properties in cell communication. Imbalance caused by increased generation of free radicals and inefficient antioxidant capacity leads to oxidative stress with subsequent OS-derived damage to nucleic acids, lipids and proteins. Oxidative and nitrosative stress play a key role in the pathophysiology of different diseases and affects women’s reproductive health and pregnancy outcome. Albeit, the currently available studies support the concept that oxidative stress and OS-mediated damage is implicated as an essential factor in the etiology of RPL, exact mechanisms of this interaction remains largely indefinable. Future studies are required to clarify the limits of the redox window above which ROS become damaging, regulators of placental vascularization and angiogenesis, redox adaptation of the placenta to ensure positive pregnancy outcome, altered expression of antioxidant genes and underlying mechanisms through which OS affects pregnancy outcome. Exogenous sources of ROS production such as smoking and alcohol consumption are important contributors of oxidative stress and hold the potential for negative impact in maternal and fetal health. Dietary antioxidants are crucial in maintaining healthy living but data on clinical benefit or positive effects of dietary antioxidant supplementation in pregnancy and female reproductive disease are scarce. Therefore, future research in this field can provide new insights regarding the potential applications of antioxidant therapy and their role in the prevention and treatment of pregnancy complications and recurrent pregnancy loss.

## Data Availability

Medline and Cochrane databases.

## Abbreviations

RPL: Recurrent pregnancy loss
ART: Assisted reproductive technology
APS: Antiphospholipid syndrome
ETC: Electronic transport chain
ROS: Reactive oxygen species
RNS: Reactive nitrogen species
MnSOD: Manganese superoxide dismutase
Cu/ZnSOD: Copper/zinc superoxide dismutase
NADPH: Nicotinamine adenide dinucleotide phosphate
CAT: Catalase
GPx: Glutathione peroxidase
GR: Glutathione reductase
GOx: Glutathione oxidase
UV: Ultra violet
GSH: Glutathione
GSSG: Oxidised glutathione
MDA: Malondialdehyde
H_2_O_2_: Hydrogen peroxide
OH^−^: Hydroxyl radicals
ROO^−^: Peroxyl radicals
PC: Protein carbonyl
8-OHG: 8-Hydroxyguanosine
8-OHdG: 8-Hydroxydeoxyguanosine
SOA: Superoxide anion
TAC: Total antioxidant capacity
TOL: Total oxidant level
NO: Nitric acid
LPO: Lipid peroxidation
PUFA: Polyunsaturated fatty acids
OS: Oxidative stress
NS: Nitrosative stress
aCL: Anticardiolipin antibodies
Se: Selenium
Zn: Zinc
Cu: Cooper
Mn: Manganese
GST: Glutathione S-transferase
NAC: *N*-Acetylcysteine
FGF: Fibroblast growth factor
VEGF: Vascular endothelial growth factor
VEGFR: Vascular endothelial growth factor receptor
N_2_O: Nitrous oxide
NO3^−^: Peroxynitrite
HNO_3_: Peroxynitrous acid
NOS: Oxide synthase
